# Resection arthroplasty for luxation of the manubrio-sternal joint in rheumatoid arthritis—a case report

**DOI:** 10.3109/17453674.2010.480940

**Published:** 2010-05-21

**Authors:** Marloes W J L Schmitz, Maarten C de Waal Malefijt, Hedwig A E M van Heereveld

**Affiliations:** ^1^Department of Orthopaedics; ^2^Department of Rheumatology, Radboud University Nijmegen Medical Centrethe Netherlands

## Introduction

In 2001, we saw a 54-year-old woman with destructive seronegative rheumatoid arthritis (RA). Because of secondary osteoarthritis, she had received a replacement of the right hip and of both knees, and a triple arthrodesis of the right foot. Furthermore, she had chronic obstructive pulmonary disease (COPD) and bronchiectasia.

She complained of severe pain and a sensation of heavy pressure at the site of the manubrio-sternal joint (MSJ), which had developed at the beginning of 2000, making coughing very difficult. She had a tender swelling at the same location on the sternum and we saw a dorsal dislocation of the manubrium. A severe thoracic kyphosis was also observed. A radiograph and CT showed a luxation of the MSJ (Figure). The patient also required a hemiarthroplasty of the left shoulder, so we planned to perform an arthrodesis of the MSJ in the same session.

**Figure:**
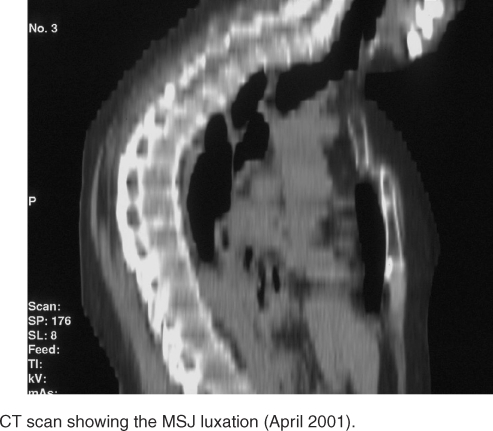
CT scan showing the MSJ luxation (April 2001).

Hemiarthroplasty of the left shoulder was first performed and then the arthrodesis of the sternum was started. However, during this procedure, on trying to place the manubrium back into position, we observed that there would still be a bone gap of about 1 cm and too much tension on the manubrium. We decided, therefore, to do a resection arthroplasty. Approximately 1 cm of the manubrium and 1 cm of the sternum was resected and the gap was closed by the soft tissue layers, which had been displaced. The skin was closed and a deep drain was left in.

During the first 2 years after surgery, she had no pain or sensation of pressure on the sternum, and the swelling had almost disappeared. She could sit in a more relaxed position, which made coughing easier. Later on, the swelling slowly progressed but the patient still did not experience any pain. At 7 years, physical examination revealed an eminent swelling of the sternum but without any tenderness. The patient was very satisfied with the result. Radiography showed that the sternum was positioned 4 cm anteriorly to the manubrium.

## Discussion

In 1950, Bogdan and Clark first described 5 cases of painful rheumatoid involvement of the MSJ. In 2 cases, the symptoms disappeared spontaneously after just 1 month. In 1 case, the pain was relieved by aspirin and in the other 2 cases no treatment was mentioned. In 1979, Rapoport et al. (1979) described an RA patient with a painful anterior subluxation of the sternal body on the manubrium. No treatment was discussed, however. In 1981, Wiseman described a patient with rheumatoid arthritis with posterior dislocation of the manubrium on the sternum, and pointed out that complaints in this area could be the result of a dislocation.

Khong and Rooney (1982) reviewed approximately 400 patients with classical RA. 10 of them had a subluxation of the MSJ, all of whom had substantial swelling over the MSJ with crepitus and deformity. Erosions and subluxation were found on lateral radiographs of the sternum in all 10 cases. The authors referred to Laitinen et al. (1970) and Kormano (1970) who had also reported that the MSJ is often involved in RA, but seldom leads to significant clinical problems. They did not mention the exact symptoms of the patients with subluxation of the MSJ, and nothing was written about the patients’ desire for treatment of their symptoms, if this should be available. Rapoport et al. (1979), Holt and Rooney (1980), Wiseman (1981), Khong and Rooney (1982), and Kelly et al. (1986) described 12 RA patients in total with thoracic kyphosis and MSJ dislocation. In the patients they described, 11 had severe kyphosis—as in our patient. Kelly et al. (1986) described the anatomical development of MSJ as the reason that it can be involved in RA, and described the role of kyphosis in transmitting force via the first rib to the manubrium, which can lead to dislocation.

We have not found any reports on the treatment of symptomatic MSJ luxation.
